# Tree Species Composition Influences Enzyme Activities and Microbial Biomass in the Rhizosphere: A Rhizobox Approach

**DOI:** 10.1371/journal.pone.0061461

**Published:** 2013-04-18

**Authors:** Shengzuo Fang, Dong Liu, Ye Tian, Shiping Deng, Xulan Shang

**Affiliations:** 1 College of Forest Resources and Environment, Nanjing Forestry University, Nanjing, People’s Republic of China; 2 Department of Plant and Soil Sciences, Oklahoma State University, Stillwater, Oklahoma, United States of America; Lakehead University, Canada

## Abstract

*Monoculture* causes nutrient losses and leads to declines in soil fertility and biomass production over successive cultivation. The rhizosphere, a zone of usually high microbial activities and clearly distinct from bulk soil, is defined as the volume of soil around living roots and influenced by root activities. Here we investigated enzyme activities and microbial biomass in the rhizosphere under different tree compositions. Six treatments with poplar, willow, and alder mono- or mixed seedlings were grown in rhizoboxes. Enzyme activities associated with nitrogen cycling and microbial biomass were measured in all rhizosphere and bulk soils. Both enzyme activities and microbial biomass in the rhizosphere differed significantly tree compositions. Microbial biomass contents were more sensitive to the changes of the rhizosphere environment than enzyme activities. Tree species coexistence did not consistently increase tested enzyme activities and microbial biomass, but varied depending on the complementarities of species traits. In general, impacts of tree species and coexistence were more pronounced on microbial composition than total biomass, evidenced by differences in microbial biomass C/N ratios stratified across the rhizosphere soils. Compared to poplar clone monoculture, other tree species addition obviously increased rhizosphere urease activity, but greatly reduced rhizosphere L-asparaginase activity. Poplar growth was enhanced only when coexisted with alder. Our results suggested that a highly productive or keystone plant species in a community had greater influence over soil functions than the contribution of diversity.

## Introduction

Nutrient availability in the rhizosphere is governed by soil properties, plant nutrient uptake characteristics, and interactions of plants, microorganisms, and their surrounding soil environment. Growing evidence suggests that plant species have significant impacts not only on soil physicochemical properties and organic matter quality but also on the abundance and composition of soil microbial community [1]–[3]. It is well established that microbial abundance, composition and diversity can fundamentally alter soil processes that lead to changes in soil nutrient availability [4], [5].

The rhizosphere is known to be a hot spot of plant-microbial interactions and a driving force of soil processes. Plant species could affect quantity and quality of carbon resources in the rhizosphere, which would influence the composition and diversity of microbial community in these environments [6]–[8]. Different plant species can promote proliferation of different microbial communities by releasing different amount and types of root exudates [9], [10]. Coexistence of multiple plant species may enhance the complexity of soil microorganisms by increasing the heterogeneity of root exudates and carbon that are contributed from roots and decomposing litter [11], [12]. Moreover, plants may directly or indirectly impact soil nutrient availability by influencing soil enzyme activities through releasing extracellular enzymes and/or altering microbial community that is known to be major contributors of enzyme activities in soil [13]–[15]. It has been reported that plants have a major influence in shaping rhizosphere microbial communities when they are grown in monoculture [6], [16]–[18]. However, the effects of plant species coexistence on soil microbes and soil enzyme activities are less known. Some studies reported herbaceous plant species coexistence did not significantly affect soil microbial biomass C, but increased microbial functional group diversity index and enzyme activity under contaminated soils [15], [19]. Mao et al. [20] suggested that mixing N-fixing shrub species into poplar stands can improve metabolic quotient and soil fertility and increase productivity in a long run. However, few studies have been conducted to elucidate effects of woody plant coexistence on rhizosphere microbial community and enzyme activities and the relationships between below-ground soil biology and above-ground plant growth characteristics.

Microbial characterization of tree rhizosphere provides vital information relating to evaluating soil nutrient status [18], [21]. However, rhizosphere studies have been challenged by the lack of a satisfying method for obtaining sufficient soil samples for subsequent laboratorial analysis. Several procedures based on shaking or washing-off soil particles adhering to roots have been proposed for the separation of rhizosphere soil from bulk soil [22]–[24]. However, it is known that soil texture and moisture strongly influence the amount of soil adhering to the root system, and root induced changes on some soil variables have been observed up to about 7 mm from the surface of an active root segment or a root mat [25], [26]. Therefore, rhizosphere studies often involve sampling procedures that permit evaluation of this gradient variation, making it challenging for data interpretation and comparison of results from different experiments.

*Fast-growing plantations, such as poplar,* are playing an increasingly important role in ameliorating environments and facilitating socio-economic sustainable development [27], [28]. However, monoculture plantations lead to increasing challenges in pest control and declines in soil fertility and plant biomass productivity over successive rotations [20], [29]. Therefore, mixed plantations have been recommended to combat insect damages and soil degradation while maintaining or increasing stand productivity [30], [31].

Much of the past research efforts were devoted to understanding crop production ecosystems, with less known on the impact of tree compositions and species coexistence on soil nutrient cycling and seedling growth [5]. In this study, we conducted a greenhouse experiment to evaluate the effect of monoculture and mixed plantations of three tree species on rhizosphere enzyme activities and microbial biomass content. The specific objectives were to: (1) assess the effects of tree species compositions on enzyme activities and microbial biomass in rhizospheric and non-rhizospheric soils and (2) investigate the relationships between enzyme activities and nitrogen (N) cycling and microbial biomass in rhizospheric soils.

## Materials and Methods

### Ethics Statement

No specific permissions were required for sampling the soil in the study area because the location is not privately-owned or protected in any way and there are no endangered or protected species in the soil sampled.

### Rhizobox Design and the Planting Experiment

**Figure 1:**
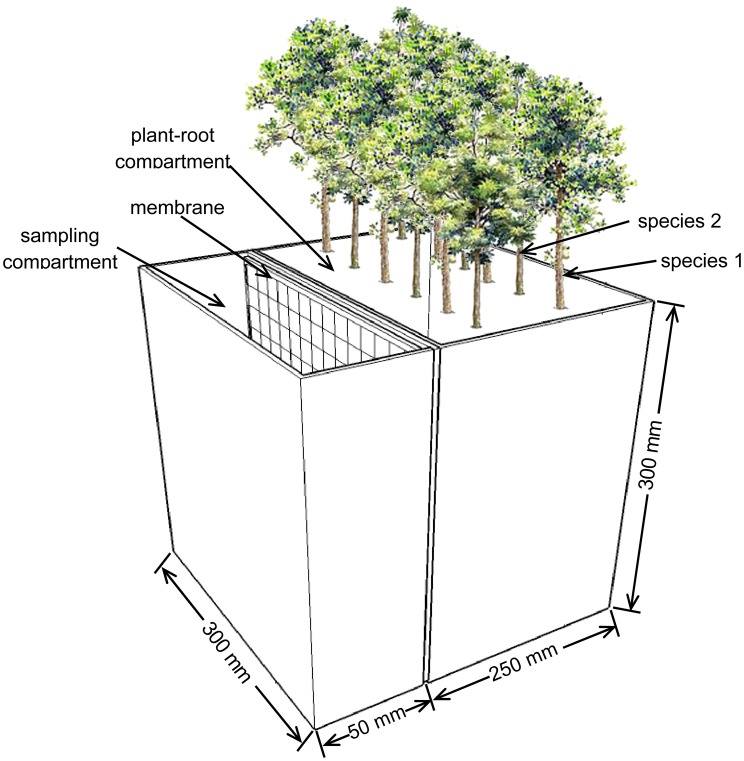
Schematics of the rhizobox used in the experiment. The rhizobox is 300×300 mm (depth ×width) with an internal volume of 22.5 L in the plant-root compartment and 4.5 L in the sampling compartment.

The rhizoboxes were made of two top-open black-plexiglas compartments separated by a polyamide membrane that has 30 µm pore-diameter and is 75 µm thick (Fig. 1). The plant-root compartment was 250 mm wide and had an internal volume of 22.5 L. The sampling compartment, where rhizosperic and bulk soils were sampled, was 50 mm wide and had an internal volume of 4.5 L. The soil used was taken from 5–15 cm depth of flooded area along Yangtze River near Maanshan City in Anhui Province (N31°41′, 118°26′E). This soil has pH of 7.2 and organic carbon of 19.0 g kg^–1^, total nitrogen of 1.93 g kg^–1^, total phosphorus of 0.40 g kg^–1^, and total potassium of 6.96 g kg^–1^. The two compartments of the rhizoboxes were packed with the soil at bulk density of 1.05 g cm^–3^ after the soil was sieved through a 2 mm mesh.

Three tree species that are commonly found in seasonal flooding areas and six compositions of these species were evaluated. The species included poplar (*Populus × euramericana* cv. ‘Nanlin-895’), willow (*Salix* × *jiangsuensis* cv. J799), and alder (*Alnus trabeculosa*). The six tested treatments (planting patterns) included poplar (P), willow (S), alder (A), poplar-willow (P×S, 1∶1), poplar-alder mixture (P×A, 1∶1), and poplar-willow-alder mixture (P×S×A, 1∶1∶1). The unrooted cuttings of poplar and willow, and 1-year-old bare seedlings of alder were planted in March 2010. The cutting length for the two species was 15–18 cm, while the cutting diameters for poplar and willow were 1.0–1.2 cm and 0.6–0.8 cm, respectively. During the planting, heights of alder seedlings ranged from 30 to 35 cm and basal diameter was from 0.3 to 0.5 cm, with intact root systems. In the plant-root compartment of each rhizobox, 12 trees were planted in two rows that were spaced 5 cm × 10 cm between plants and 2.5 cm or 7.5 cm from the compartment edges. For treatments employed more than one species, different tree species were randomly placed in alternative spots within rows (see Fig. 1). Each treatment was replicated three times. Plants were grown in a greenhouse with night and day temperatures of 10 and 35°C and soil water content maintained at 60% water holding capacity. During the 8-month growing period, roots penetrated soil in the plant-root compartments, and root mats were formed along the membranes, which were in contact with soil in the rhizosphere compartments.

### Soil Sampling and Preparation

After eight months of growth in the rhizobox (November, 2010), heights and basal diameters of all planted trees were measured; the two compartments of each rhizobox were separated carefully. In the sampling compartments, soils in contact with root mats were sliced vertically into three sequential sections of approximately 2 mm, 2 mm, and 6 mm width and sampled as rhizosphere soils, while the soils at the distance of 10–50 mm to the root mats were sampled as bulk soils (non-rhizosphere soils). Each sample was mixed thoroughly, sieved through a 2-mm mesh, and then divided into two portions. One was air dried for chemical analysis, and the other was stored at 4°C before subsequent analysis of microbial biomass and enzymatic activities.

### Enzymatic Activity Assays

Enzyme activities associated with N cycling, including L-asparaginase, urease, and proteinase, were evaluated. L-asparaginase activity was determined as described by Frankenberger and Tabatabai [32] and was expressed as mg NH_4_^+^–N released g^–1^ soil day^–1^. Briefly, 5 g of soil was incubated with buffered (0.1 M THAM, pH 10) L-asparagine at 37°C for 0.5 h, followed by the quantification of NH_4_^+^ released. Urease and proteinase activities were determined in triplicates at optimal pH for each enzyme according to methods described by Liu et al. [18]. Urease activity was expressed as mg NH_3_^+^–N released g^−1^ soil day^−1^, while proteinase activity was expressed as mg tyrosine released g^−1^ soil day^−1^.

### Microbial Biomass Measurements

Fumigation and microbial biomass was measured following the procedure described by Joergensen and Brookes [33]. Each fresh soil (5 g on an oven-dry basis) was fumigated with ethanol-free CHCl_3_ for 24 h. After adding 0.5 M K_2_SO_4_ (4∶1 solution to soil ratio), the soil was shaken for 30 min on an oscillating shaker. The suspension was centrifuged, and then the supernatant was filtered through a Whatman polyethersulfone syringe filter (pore size: 0.45 µm). Non-fumigated soil was used as a control. All results are expressed on an oven-dry soil basis (105°C, 24 h) and are means of triplicate laboratory analyses of samples from three replicate treatments.

Microbial organic C (MOC) in the K_2_SO_4_ soil extracts was measured by an automated uv-persulphate oxidation method [34]. Microbial biomass C (MBC) was calculated from MBC = 2.22 MOC, where MOC = [(C extracted from fumigated soil)-(C extracted from non-fumigated soil)] [34]. Ninhydrin–N in the K_2_SO_4_ soil extracts was measured according to the method described by Joergensen and Brookes [33] and was used to calculate microbial biomass N (MBN) (MBN = 5.0 ninhydrin–N) [33], [35].

### Statistical Analyses

Statistical analyses were carried out using SPSS 13.0 (SPSS, Chicago, IL, USA). Both one-way and two-way ANOVA were conducted to compare enzymatic activities and microbial biomass in the rhizosphere and non-rhizosphere soils among tree composition treatments and distances to root mats. A Duncan’s multiple range test was used to detect differences between treatment means. All statistical analyses were carried out at p<0.05. Relationships between enzyme activities and microbial biomass were evaluated using Pearson’s correlation analysis.

## Results

### Enzymatic Activities

**Figure 2:**
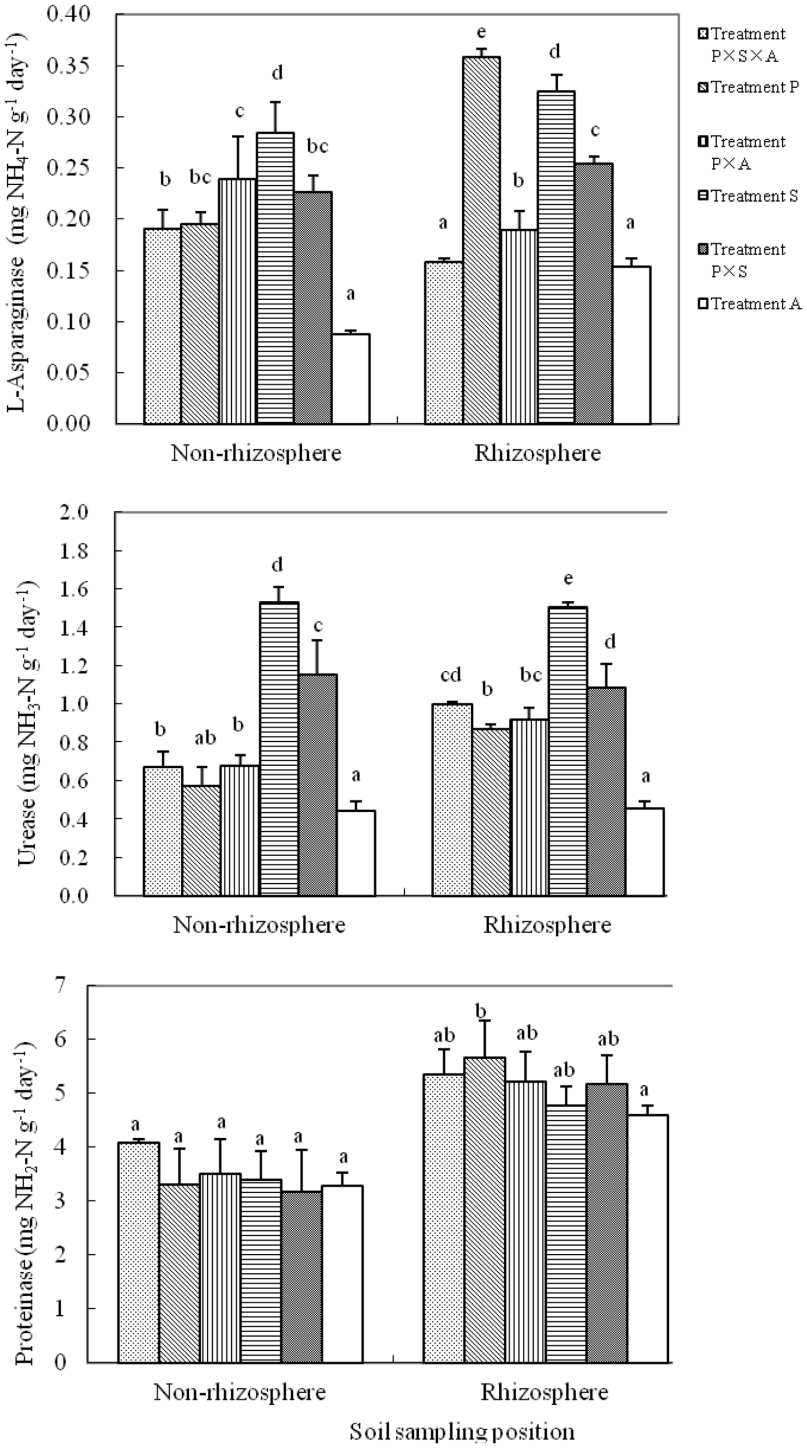
Activities (mean ± standard deviation) of L-asparaginase, urease and proteinase in the rhizosphere (0 to 10 mm to the root mats) and non-rhizosphere soils (>10 mm to the root mats) under six different tree species compositions. Bars indicate standard deviations. Treatments P, S, A, P×S, P×A and P×S×A refer to poplar, willow, alder, poplar-willow, poplar-alder, and poplar-willow-alder, respectively. Different letters indicate significantly different means within the same sampling position at P<0.05 according to Duncan’s multiple range test.

L-asparaginase urease, and proteinase in both rhizosphere and non-rhizosphere soils were significantly affected by tree species compositions (p<0.05), with the exception of proteinase activity in non-rhizospere soils (Fig. 2). In the rhizosphere, L-asparaginase varied from 0.15 to 0.36 mg NH_4_^+^–N g^−1^ day^−1^, with the highest activity in treatment P (poplar monoculture), followed by treatment S (willow monoculture), and the lowest in treatment A (alder monoculture). L-asparaginase activity in the monoculture of poplar or willow was significantly higher than that in the mixed culture of poplar and willow (Fig. 2). Compared to poplar monoculture, L-asparaginase activity in treatment P×S×A (poplar-willow-alder coexistence), treatment P×A (poplar-alder coexistence) and treatment P×S (poplar-willow coexistence) was reduced by 126.1%, 98.1% and 51.6%, respectively. The trend of L-asparaginase activity in the non-rhizosphere soils of different planting patterns was somewhat different from that found in the rhizophere soils. The highest L-asparaginase activity in the non-rhizosphere soils was observed in treatment S, followed by patterns 3, 5, 2, and then treatment P×S×A, with the lowest found in treatment A (Fig. 2).

The rhizosphere urease activity ranged from 0.45 to 1.51 mg NH_3_^+^–N g^−1^ day^−1^, with the highest in treatment S, followed by treatment P×S, and the lowest in treatment A (Fig. 2). Compared to poplar monoculture, other species addition significantly increased rhizosphere urease activity, where the urease activity in treatment P×S×A, treatment P×A and treatment P×S was 15.1%, 5.8% and 25.3% higher than the treatment P, respectively. Similar trend was observed in the non-rhizosphere soils (Fig. 2).

Trend for proteinase activities was somewhat different from those for activity of urease or L-asparaginase. Activity of proteinase was significantly higher in the rhizosphere soils than in non-rhizosphere soils, and planting patterns showed limited impact on its activities (Fig. 2). Proteinase activity ranged from 4.60 to 5.66 mg NH_2_–N g^–1^ day^−1^ in the rhizosphere soils and 3.18 to 4.09 mg NH_2_–N g^−1^ day^−1^ in the non-rhizosphere soils (Fig. 2). In the rhizosphere soils, the highest proteinase activity was found in treatment P, and the lowest in treatment A. However, proteinase activity in the non-rhizosphere soils was not significantly different among different planting patterns.

**Figure 3:**
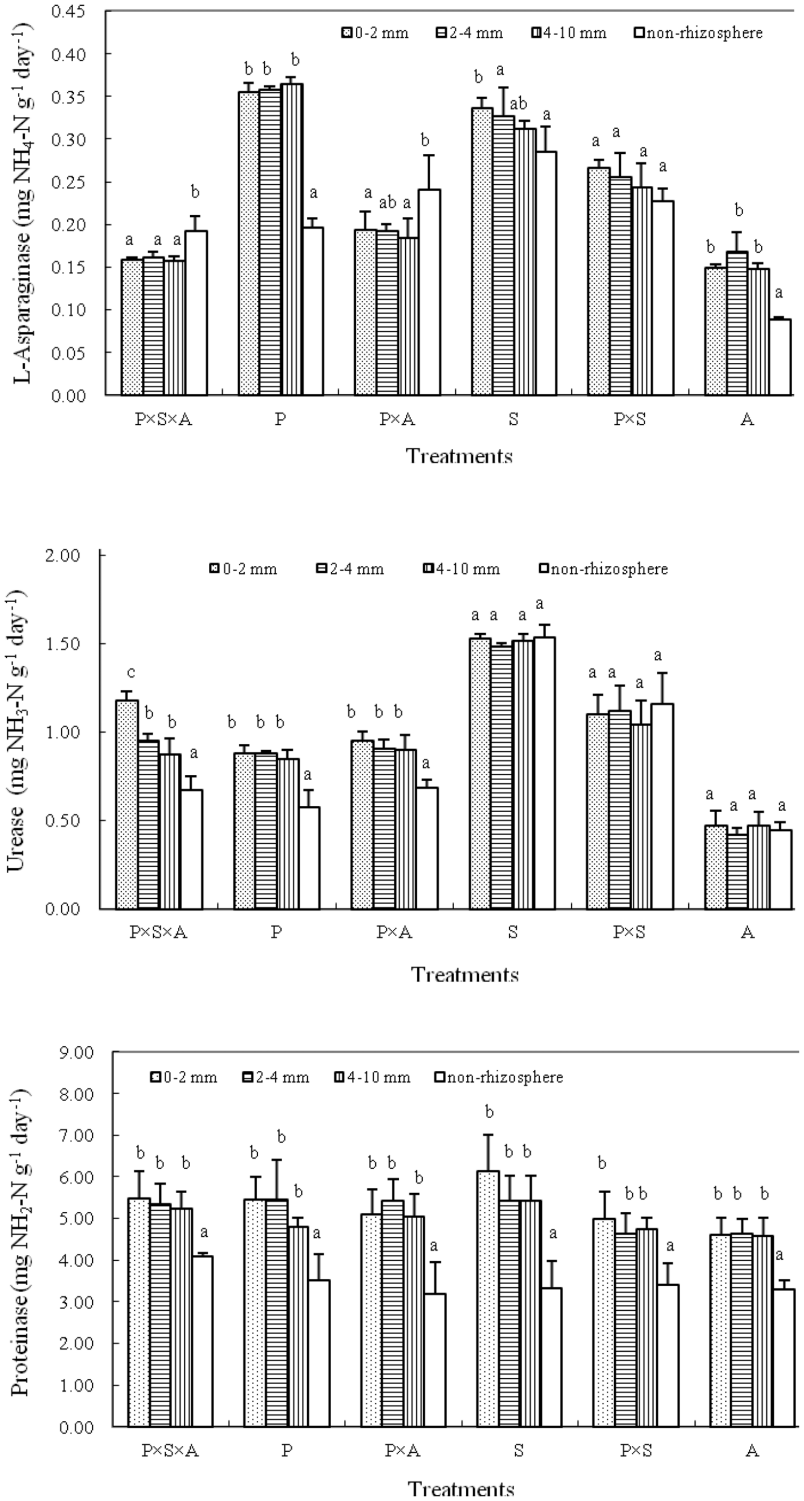
Activities (mean ± standard deviation) of L-asparaginase, urease and proteinase in soils at different distances to the root mats under different tree species compositions. Bars indicate standard deviations. Treatments P, S, A, P×S, P×A and P×S×A refer to poplar, willow, alder, poplar-willow, poplar-alder, and poplar-willow-alder, respectively. Different letters indicate significantly different means within the same treatment at P<0.05 according to Duncan’s multiple range test.

The spatial variation of enzyme activities in the rhizosphere is showed in Fig. 3. In most cases, no significant difference was detected for tested enzymes within 10 mm to the root mats in the rhizosphere soils. However, the rhizosphere L-asparaginase activity in treatment S and the urease activity in treatment P×S×A were significantly different among the three distances to the root mats within the rhizoshpere, showing an increasing tendency as approaching the root mats (Fig. 3).

### Microbial Biomass

**Figure 4:**
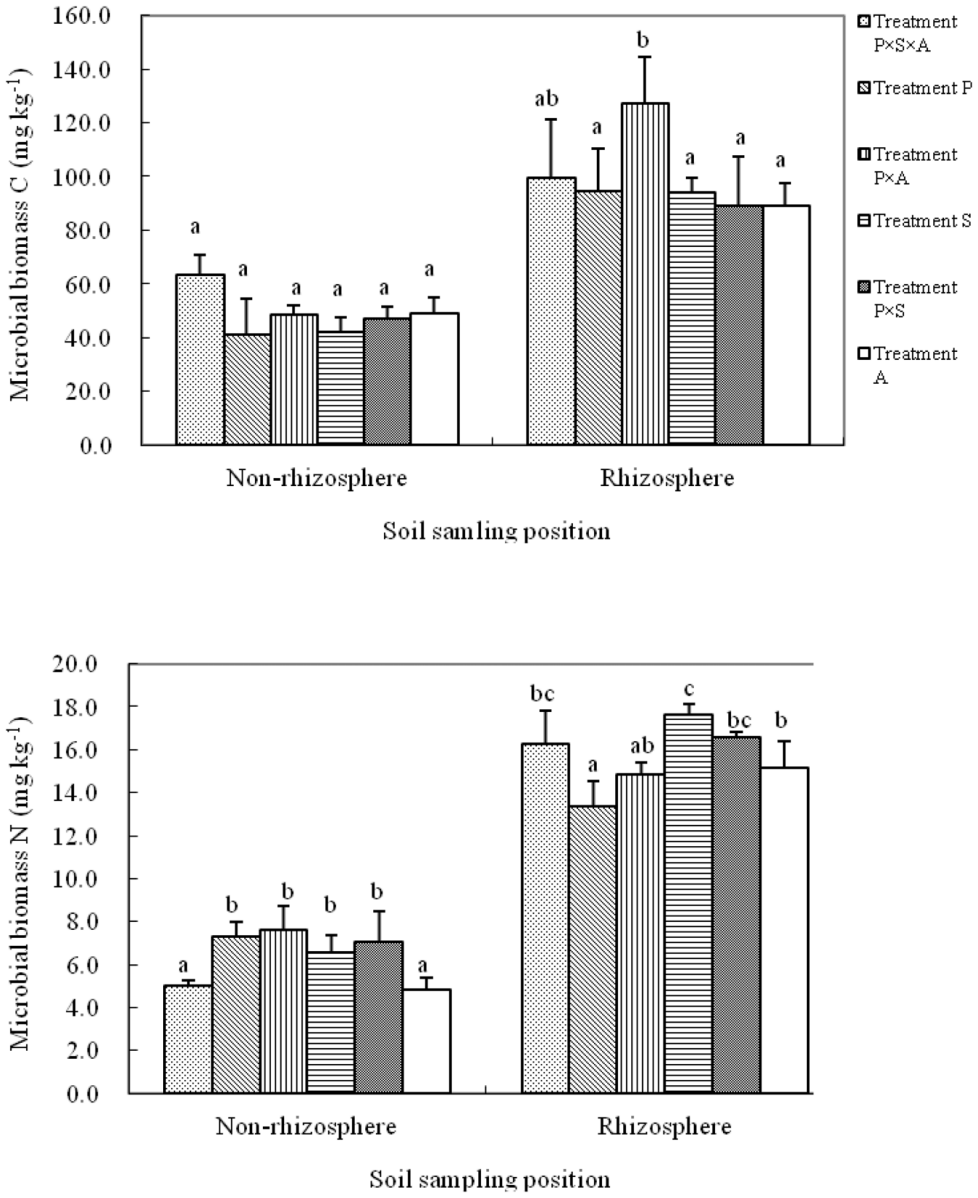
Content of microbial biomass C and N in the rhizosphere and non-rhizosphere soils under different tree species compositions (means ± standard deviation). Treatments P, S, A, P×S, P×A and P×S×A refer to poplar, willow, alder, poplar-willow, poplar-alder, and poplar-willow-alder, respectively. Different letters indicate significantly different means within the same sampling position at P<0.05 according to Duncan’s multiple range test.

Soil MBC and MBN varied significantly in the rhizosphere soils among different planting patterns (Fig. 4). The highest MBC was observed in treatment P×A, reaching 127.3 mg kg^–1^. The MBC value in treatment P×A was significantly greater than the values in patterns 2, 4, 5 and 6, but was slightly higher than the value in treatment P×S×A. Compared to poplar monoculture, alder addition increased rhizosphere MBC in poplar mixtures, e.g. increasing 5.6% in treatment P×S×A and 34.6% in treatment P×A, respectively. However, the greatest MBN was measured in treatment S, followed by treatment P×S and the lowest was found in treatment P (Fig. 4). Compared to poplar monoculture, other species addition significantly increased rhizosphere MBN, where the MBN in treatment P×S×A, treatment P×A and treatment P×S was 21.6%, 11.2% and 23.9% higher than the treatment P, respectively.

Tree species compositions did not significantly affect MBC in the non-rhizosphere soils (Fig. 4). However, a significant difference in MBN among the planting patterns was observed, with treatment P×S×A and 6 being significantly lower than those found in other tested patterns (Fig. 4).

Comparing to MBC at 4–10 mm distance, its content at 0–2 mm was about 1.3–1.5 fold in mixed planting patterns, and 1.1–1.4 fold in monoculture. However, the MBN values at 4–10 mm distance in willow monoculture and poplar-willow mixture) was 28.1% and 5.1% higher than at 0–2 mm distance respectively, though the MBN at 0–2 mm distance increased by a factor of 1.1–1.6 in the other planting patterns compared to the distance of 4–10 mm.

The spatial variation of MBN and MBC in both rhizosphere and non-rhizosphere soils is showed in Table 1. For all planting patterns, MBN and MBC contents were significantly higher in rhizosphere than non-rhizosphere soils. In most cases, a significant difference in MBN and MBC was detected among the three distances to the root mats in the rhizosphere soils, except for MBN in treatment P×A and MBC in treatment P and treatment S (see Table 1). The mean MBN in the rhizosphere, where treatments with six planting patterns averaged within distance classes, was 16.5±0.8, 15.7±2.4 and 14.8±3.3 mg kg^−1^ at distances of 0–2, 2–4 and 4–10 mm to the root mats, respectively, while the mean MBC was 112.5^b^±15.2, 98.9^ab^±19.3 and 85.8^a^±12.3 mg kg^–1^ (where different superscript letters indicate significant difference at p<0.05).

**Table 1:** Gradient variation of microbial biomass nitrogen (N) and carbon (C) content (mg kg^−1^) in the rhizosphere of different tree species compositions.†

Index	Tree species composition (Planting patterns)	Microbial biomass N or C content in soils at distance to the root mats (mm)			
		0–2	2–4	4–10	10–50
Microbial biomass N	Poplar (treatment P)	17.0±1.6c	12.3±1.4b	10.9±1.5b	7.3±0.7a
	Willow (treatment S)	16.0±0.3b	16.5±1.1b	20.5±0.5c	6.6±0.8a
	Alder (treatment A)	17.5±0.8c	13.9±1.5b	14.2±1.6b	4.8±0.6a
	Poplar-willow (treatment P×S)	15.6±0.9b	17.9±0.8c	16.4±0.9bc	7.1±1.4a
	Poplar-alder (treatment P×A)	15.7±1.2b	15.1±0.9b	13.8±0.9b	7.6±1.1a
	Poplar-willow-alder (treatment P×S×A)	17.1±1.5c	18.7±1.5c	13.1±1.8b	5.0±0.2a
Microbial biomass C	Poplar	107.3±21.6b	99.3±11.5b	77.1±13.7b	41.5±12.9a
	Willow	100.3±3.4b	92.5±1.1b	89.3±9.9b	42.1±5.4a
	Alder	98.3±3.4c	82.3±9.1b	86.7±5.3bc	49.4±5.7a
	Poplar-willow	108.2±0.41c	87.1±5.9b	72.9±15.7b	47.0±4.6a
	Poplar-alder	137.9±17.5c	136.3±8.9c	107.7±12.6b	48.8±3.1a
	Poplar-willow-alder	123.0±21.0c	95.6±9.4b	81.1±7.5b	53.6±7.3a

† Data represent means ± standard deviation. Different lower case letters indicate significantly different means within the row at P<0.05 according to Duncan’s multiple range test.

### Microbial C/N Ratio

**Figure 5:**
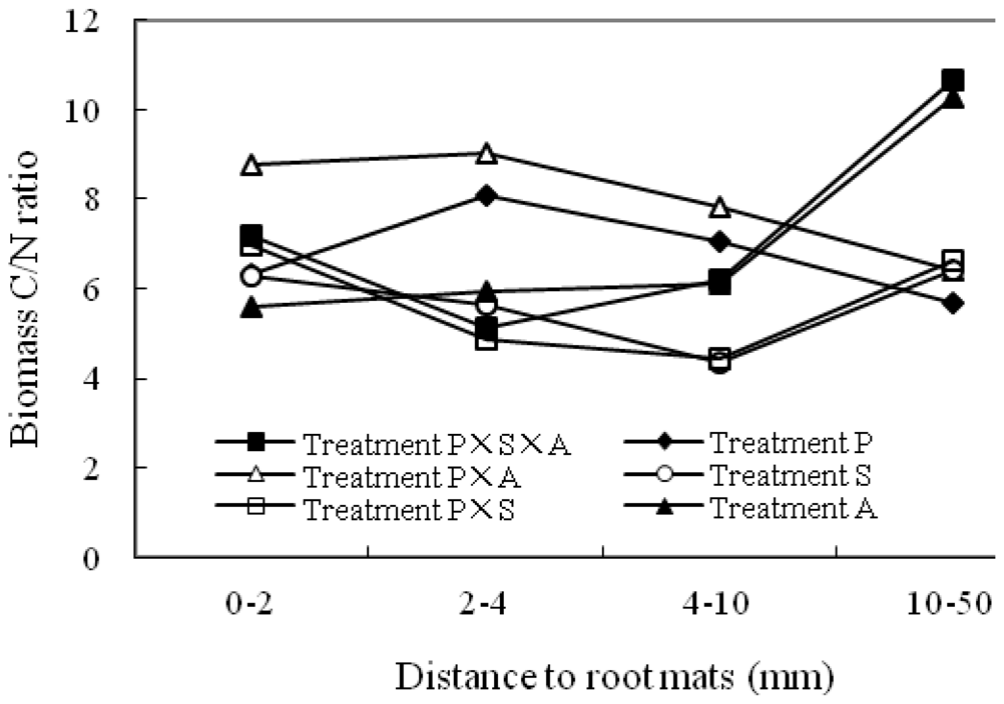
Ratios of microbial biomass C and N content in soils at different distances to the root mats under different tree species compositions. Treatments P, S, A, P×S, P×A and P×S×A refer to poplar, willow, alder, poplar-willow, poplar-alder, and poplar-willow-alder, respectively.

**Figure 6:**
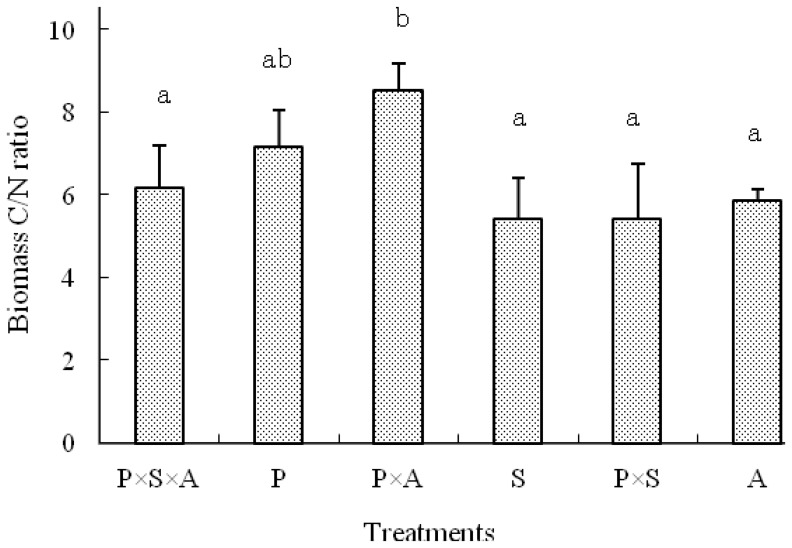
Ratios of microbial biomass C and N content in the rhizosphere soils under different tree species compositions (means ± standard deviation). Treatments P, S, A, P×S, P×A and P×S×A refer to poplar, willow, alder, poplar-willow, poplar-alder, and poplar-willow-alder, respectively. Means with the same letter are not significantly different at P<0.05 according to Duncan’s multiple range test.

The microbial C/N ratio in the rhizosphere ranged 5.6–8.6, 4.9–9.0, 4.4–7.8 at the distances of 0–2, 2–4 and 4–10 mm to the root mats, respectively, while in the non-rhizosphere was from 5.7 to 10.7 (Fig. 5). The mean C/N ratios across six planting patterns within distance classes, was 6.9±1.1, 6.4±1.7, 6.0±1.4 and 7.7±2.2 at 0–2, 2–4, 4–10 and 10–50 mm (non-rhizosphere soil) to the root mats respectively, and the observed variations between the distance to the root mats were not statistically significant (p = 0.365). However, the results demonstrated that the microbial C/N ratio in the rhizosphere was significantly influenced by the planting patterns (Fig. 6). The highest C/N ratio was achieved in treatment P×A, which is 38.3, 19.3, 57.5, 57.4 and 45.1% greater than those in patterns 1, 2, 4, 5 and 6, respectively.

**Figure 7:**
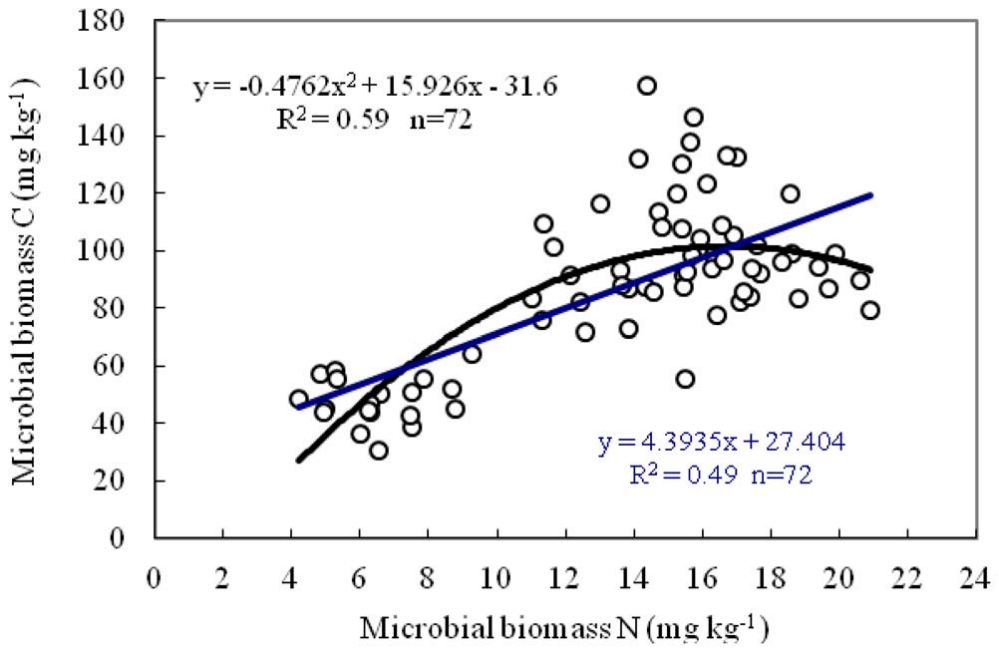
Relationships between microbial biomass C and N content in all soils tested (n = 72).

Furthermore, a significant correlation existed between soil microbial biomass C and N (Fig. 7), and R^2^ reached 0.59 and 0.49 for Quadratic and linear equation, respectively (n = 72, p<0.001).

### Seedling Growth

Seedling height and basal diameter growth of three tree species were affected by planting patterns (Table 2). At the end of the 8-month growing period, the highest increment of height and basal diameter for poplar and willow were not found in monoculture but mixed planting patterns. Poplar grew best in mixed culture with alder (treatment P×A), while willow in both mixed planting patterns. Alder grow best in monoculture, but its seedling height and basal diameters were not significantly higher than those in poplar-alder mixed planting patterns. Although willow grew best when planted with other two tree species, its presence led to low growth increments for poplar and alder. Compared to poplar monoculture (treatment P), annual increment of height in treatment P×A increased by 18.3%, whereas decreased by 30.9% and 30.8% in patterns 1 and 5, respectively. Similar trend was found in basal diameter increment.

**Table 2:** Tree growth in eight months under various planting patterns.†

Planting patterns	Net height increment (cm)			Net basal diameter increment (cm)		
	poplar	willow	alder	poplar	willow	alder
Poplar-willow-alder	82.9±13.5a	170.8±20.3a	44.1±3.8a	0.65±0.22a	0.70±0.13ab	0.20±0.06a
Poplar-willow	83.1±10.5a	178.1±1.4a	/	0.58±0.08a	0.71±0.02b	/
Poplar-alder	141.9±6.8b	/	58.9±7.8b	0.96±0.05b	/	0.26±0.05a
Poplar	120.0±14.8b	/	/	0.91±0.11b	/	/
Willow	/	164.1±6.1a	/	/	0.53±0.06a	/
Alder	/	/	60.3±2.9b	/	/	0.33±0.06a

† Data represent means ± standard deviation. Different letters indicate significantly different means among the planting patterns at P<0.05 according to Duncan’s multiple range test.

## Discussion

### Tree Species Coexistence and Microbial Activity

In natural ecosystems, plant diversity influences the composition and biomass of microbial communities [36], [37] because rhizodeposits or organic compounds released by plants can be highly specific for a given plant species or even a particular cultivar [17], [38]. However, interactions between plant species and soil organisms have been reported to be context dependent [39] or even lack such relationships [17], [40], [41]. Results from the present study indicated that rhizosphere microbial activity was significantly affected by the tree species compositions, but the highest L-asparaginase, urease and proteinase as well as the greatest MBC value were observed in monoculture except that the highest MBN was achieved in the poplar-alder mixture (Fig. 2 and Fig. 4). Our results support the idea that a highly productive or keystone plant species present in the community results in greater influence over soil functions than the contribution of additional diversity per se [2], [42], [43], but in contrast to the idea that a higher diversity will have higher functioning by complementarity of species traits [42], [44].

Data from this study indicated that composition and complementarity of species traits are also important factors influencing rhizosphere microbial community and functionality. This influence is, in part, evidenced by differences in microbial biomass C/N ratios at different distances from the root mats or among different planting patterns. Different microbial biomass C/N ratios signify changes in microbial community composition. Marilley and Aragno [45] revealed that members of the Cytophaga-Flexibacter-Bacteriodes phylum were ubiquitous in the bulk and rhizosphere soils of *Trifolium repens* and *Lolium perenne*. In fact, most soil microbes appear to be ubiquitous [46]. Based on PCR-DGGE detection, Kandeler et al. [47] showed that about 10% of the detected bacteria in the rhizosphere of maize were rhizosphere-specific. Nevertheless, the impact of plants to rhizosphere microbial community was detectable. Soil microbial C/N ratios often range between 5 to 8 [48]. In this study, microbial biomass C/N ratios were as high as about 11 under poplar-willow-alder or alder monoculture, suggesting that these rhizopheres promoted proliferation of actinomycetes and fungal community. All the high microbial biomass C/N ratios detected in this study were under alder mono- or mixed culture. It seems that alder could be playing a vital role inducing the observed higher microbial biomass C/N ratios. This coincided to certain degree with trend observed on seedling growth. Alder grew best in monoculture, while poplar grew best in mixed culture with alder. Further studies are needed to elucidate factors that induced the observed changes of microbial biomass to significantly higher C/N ratios than commonly observed in soil and relationship of these change to seedling growth. However, when included all data in the analysis, microbial biomass C/N ratio was about 4.4, which clearly indicate dominance of bacteria in the soil environment.

### Relationship between Enzyme Activity and Microbial Biomass

A positive correlation between tested enzyme activity and microbial biomass was observed in this study (Table 3), but correlation coefficients were very different for the specific enzyme activity. Proteinase activity was significantly and positively correlated with MBC (r = 0.822, p<0.01) and MBN (r = 0.765, p<0.01), indicating that proteinase activity was associated with active microorganisms in the soil that are the major source of soil enzymes. However, urease activity was weakly correlated with MBC and MBN, and almost no correlation was found between L-asparaginase activity and soil microbial biomass (see Table 3), supporting the idea that plants also liberate enzymes to the soil through root exudates or after the death and rupture of the cells [49]. The correlations between microbial biomass and enzyme activity are influenced by many factors [50], and results reported are also inconsistent with different researchers. For example, Böhme et al. reported that a strong correlation between the activity of proteases and soil microbial biomass C was observed in the Bad Lauchstädt soil, while little correlation found for the soil from Keszthely [51]. Okur et al. indicated MBC was significantly and positively correlated with dehydrogenase, protease, urease, and alkaline phosphatase [52]. Highly positive correlations were observed between MBC and MBN as well as between L-asparaginase and urease activities in the present study (p<0.01), in coincidence with the results from Stark et al. [50].

**Table 3:** Correlation coefficients between microbial biomass contents and enzyme activities in the soils (n = 24).

Index	Microbialbiomass C	MBN	Urease	Proteinase
Microbial biomassN (MBN)	0.754**			
Urease	0.162	0.326		
Proteinase	0.822**	0.765**	0.154	
L-asparaginase	0.011	0.118	0.590**	0.228

** Significant at P<0.01.

### Stratification of Enzyme Activity and Microbial Biomass in the Rhizosphere

Rhizoboxes have been widely used to study the rhizosphere of annual crops and young plants, but have been rarely applied to tree species [24], [53]. In the present study we assessed enzyme activity and microbial biomass gradients in the rhizosphere (defined the soil within 10 mm to the root mats) associated with different tree species compositions. Our results indicated that in most cases no significant differences were detected for tested enzymes among the three distances (0–2, 2–4 and 4–10 mm) to the root mats in rhizosphere soils (Fig. 3), and urease activity at the distance of 0–2 mm increased by a factor of about 1.3 in treatment P×S×A compared to the distances of 2–4 and 4–10 mm. The results from this study are not similar to the reported result by Kandeler et al. [47] where enzyme activities increased by a factor of approximately 2 in the first 1 mm of a maize (*Zea mays*) rhizosphere, but similar to the results *in situ* rhizobox reported by Avianil et al. [53] where the gradients of amidase activity and hydrolytic activity in fruit tree rhizosphere (within 5 mm to the root mats) were barely observed. The observed differences among different studies could be due to difference in root systems of plant species under evaluation. However, the mean enzyme activities across all planting patterns were significantly higher within 10 mm than 10–50 mm to the root mats. This suggests that root effect on enzyme activities may extend beyond 10 mm. This is evidenced by the significantly different activities of L-asparaginase and urease in the non-rhizosphere soils (Fig. 3).

On the contrary, significant differences in MBN and MBC were observed in most cases among the three distances to the root mats in the rhizosphere soils (see Table 1). Regardless of planting patterns, content of MBN and MBC decreased with increasing distance to root mats. The higher microbial biomass in the rhizosphere than bulk soil was, in part, due to migration of microbes toward growing seedlings [54] or translocated by mass flow due to moisture gradient induced by plant transpiration. The microscale variability reflected changes in substrate quantity, quality, and composition in the rhizosphere as well as sensitivity of microbial community to these changes. This is consistent with observations that rhizosphere bacterial community is predominated by r-strategists that are capable of proliferating rapidly in response to changes of substrate [47]. Generally, the values in rhizosphere gradients of microbial biomass in the present study were less than the reported results from annual crops, where microbial biomass increased by a factor of 2–4 in ryegrass [55] or up to 10 in a model rhizosphere [56] for a 1 mm distance from the root surface.

Enzyme activity and microbial biomass along rhizosphere gradient are very important information for sampling procedures of rhizosphere soil and results from different experiments should be compared carefully. Our results indicated that mean activity of 3 tested enzymes and mean MBN in the rhizosphere, where treatments with six planting patterns averaged within distance classes, were not significantly different at distances of 0–2, 2–4 and 4–10 mm to the root mats, while a significant difference in mean MBC was observed between 0–2 mm and 4–10 mm distances from root mats. Results from this study suggest that defining the soil at 0–4 mm distance from the root mats as rhizosphere soil should be more reliable for this specific rhizobox design and experiment, though sampling procedures for rhizosphere soil should depend on soil texture and structure, plant species and observed parameter, in agreement with the results from an *in situ* rhizobox approach [18].

### Conclusions

Composition and complementarity of species traits are important factors influencing rhizosphere microbial community and functionality. Both enzyme activity and microbial biomass in the rhizosphere were significantly influenced by tree species composition. However, impact of tree species coexistence on enzyme activities and microbial biomass varied, depending on complementarity of species traits. Compared to poplar monoculture, other tree species addition did not significantly affect rhizosphere proteinase activity, but led to significantly lower L-asparaginase and higher urease activity in the rhizosphere. Microbial biomass contents are more sensitive to changes in the rhizosphere environment than enzyme activities. The growth of poplar was enhanced only when coexisted with alder. In general, a highly productive or keystone plant species in a community has greater influence over soil functions than the contribution of diversity. Incorporate willow in a plantation may lead to marked increase in the capacity of soil to cycle N through activities of L-asparaginase and urease.
